# Flexible Lamination-Fabricated Ultra-High Frequency Diodes Based on Self-Supporting Semiconducting Composite Film of Silicon Micro-Particles and Nano-Fibrillated Cellulose

**DOI:** 10.1038/srep28921

**Published:** 2016-06-30

**Authors:** Negar Sani, Xin Wang, Hjalmar Granberg, Peter Andersson Ersman, Xavier Crispin, Peter Dyreklev, Isak Engquist, Göran Gustafsson, Magnus Berggren

**Affiliations:** 1 Department of Science and Technology, Linköping University, SE-601 74 Norrköping, Sweden; 2 Printed Electronics, Acreo Swedish ICT AB, Box 787, SE-601 17 Norrköping, Sweden; 3 INNVENTIA AB, Box 5604, SE-114 86 Stockholm, Sweden

## Abstract

Low cost and flexible devices such as wearable electronics, e-labels and distributed sensors will make the future “internet of things” viable. To power and communicate with such systems, high frequency rectifiers are crucial components. We present a simple method to manufacture flexible diodes, operating at GHz frequencies, based on self-adhesive composite films of silicon micro-particles (Si-μPs) and glycerol dispersed in nanofibrillated cellulose (NFC). NFC, Si-μPs and glycerol are mixed in a water suspension, forming a self-supporting nanocellulose-silicon composite film after drying. This film is cut and laminated between a flexible pre-patterned Al bottom electrode and a conductive Ni-coated carbon tape top contact. A Schottky junction is established between the Al electrode and the Si-μPs. The resulting flexible diodes show current levels on the order of mA for an area of 2 mm^2^, a current rectification ratio up to 4 × 10^3^ between 1 and 2 V bias and a cut-off frequency of 1.8 GHz. Energy harvesting experiments have been demonstrated using resistors as the load at 900 MHz and 1.8 GHz. The diode stack can be delaminated away from the Al electrode and then later on be transferred and reconfigured to another substrate. This provides us with reconfigurable GHz-operating diode circuits.

Flexible electronics have been developed since 1960 s when attempting reducing weight of solar cells by thinning Si wafer to about 100 μm^1^. Today, when not only people but also things are becoming connected to Internet, the demand for flexible electronic components is driven by a vast array of distributed applications, such as wearable electronics, e-labels and point-of-care sensors. These will serve as outposts of the Internet of Things (IoT), and to power up and communicate with all the *e*-tags a wireless energy harvesting technology that operates at high frequencies is needed. A rectifier in combination with an antenna is a viable solution for this, wherein a single diode can serve as the rectifier^2^,^3^. Diodes are also widely used in AC/DC converters, signal transmitters and receivers, voltage regulators, etc. The diode is therefore a key-enabling component for IoT solutions based on flexible electronics powered by, and communicating via, radio frequency electromagnetic waves. One way to achieve flexible circuits is to use printing methods in order to manufacture thin film devices on foils. Organic semiconductors are inherently flexible and solution-processable, and printed organic diodes and transistors have been demonstrated in the past^4^,^5^. However, achieving high carrier mobility is still a challenge for organic semiconducting materials and consequently it is difficult to reach high operating speeds and quick switching characteristics that enable high frequency performance. Mobilities up to 11 cm^2 ^V^−1 ^s^−1^ have been achieved with single crystalline organic semiconductors^5^,^6^, which still is at least one order of magnitude lower than typical mobilities found in inorganic counterparts such as silicon (Si) transistors^7^. Inorganic semiconductors, like Si, have shown superior high frequency performance and are therefore attractive in printed and/or flexible electronic circuits. Si has been the most important material for microelectronics since the solid state transistor was invented in 1947 8. The development so far has mainly been focused on reducing the dimensions of the transistor device architecture and to integrate a huge number of Si transistors into complex circuits on wafers or other planar rigid substrates^9^. Nevertheless, several methods have been explored to produce Si-based components on flexible substrates, e.g., by thinning the Si wafers or depositing Si nanomembranes to fabricate components and assembling them to a plastic substrate using lift-off, transfer printing, or peel-and-stick techniques; depositing amorphous Si on polymer substrate, turning Si wafer into Si ribbons; and using solution-processing of silane followed by post annealing^10^,^11^,^12^,^13^,^14^,^15^,^16^,^17^. However, these methods are often relatively costly, complex and require vacuum and high temperature. Printing of milled Si-μPs mixed together with a binder material offers a new but simpler route to make Si-based devices. So far, flexible Si diodes and Thin Film Transistors (TFTs) have been demonstrated^18^,^19^,^20^,^21^. Besides aforementioned organic materials and Si, other semiconductors are also incorporated in flexible, high-speed diodes^22^. ZnO nanoparticles have been utilized in printed diodes but measurements results were only presented for frequencies up to 13.56 MHz^3^. Indium–gallium–zinc–oxide has also been used to fabricate flexible UHF Schottky diodes where all the device layers are deposited via sputtering followed by photolithography patterning^23^. Comparing to standard semiconductor fab processes, printing is low cost mass production method and it brings in a new alternative way for fabricating flexible electronic components^24^. In a previous report, we have shown printed diodes using micro-sized Si particles that operate up to 1.6 GHz^18^. However, the printing process in that case involves 6 printing and 4 curing steps, requiring dedicated screen-printing and inkjet printing machines. Thus, a yet simpler method to manufacture these flexible Si-μP-based diodes is desirable^25^.

Paper-based substrates and scaffolds are composed mainly of wood fibers and are gaining increased interest from the printed electronics community, mainly because ordinary paper can be produced at low costs and large volumes, and that paper is an environmentally friendly material derived from the forest^26^. Considerable research is targeting the extraction and use of nano-fibrillated cellulose (NFC), which composes the inner structure of the wood fiber. The NFC fibers have very characteristic properties; they are extremely thin and possess a large aspect ratio (typical length up to 1 μm, diameter in the range of 5–15 nm^27^); they are hydrophilic and can be easily dispersed in water as a viscous gel; they can self-organize into thin films using casting from water solutions; and they can create very strong structures caused by the inherent mechanical properties of the cellulose. NFC does not show electronic conductivity, however, it does have a very weak ionic conductivity caused by its associated ionic charges, and it can be further functionalized by mixing it with other materials^27^,^28^.

Recently, a lamination method for the manufacturing, integration and reconfiguration of organic electronic materials, components and systems was demonstrated by using NFC as the scaffold in a thin film composite material system^29^. By mixing an NFC:glycerol suspension with either a conducting polymer (PEDOT:PSS) or a polyelectrolyte, followed by casting and drying the mixture in a petri dish, it was possible to obtain films with electronic or ionic conductivity, respectively. The dried films were self-supporting and cut pieces from the films could be assembled onto a vast array of substrates or functional layers in order to create electrochemical components, *e.g.* electrochromic displays and electrochemical transistors. The glycerol ingredient of the films provided self-adhesive properties, and their sticky surfaces made it possible to obtain good adhesion between different layers upon lamination. Furthermore, the laminated layers could be separated, cut, and reused repeatedly in various circuit configurations.

In this paper, we present a method to manufacture a “sticker label” Si-based diode for ultra-high frequency applications. NFC is here used as a binder material for the Si-μPs to create a self-supporting composite film. A Schottky contact is formed by laminating the NFC:Si-μP film onto an Al contact on a PET foil. An ohmic contact is obtained by laminating a conductive tape onto the other side of the NFC:Si film. The obtained diode rectifies AC signals all the way up to the GHz ranges. Hence, a new method to manufacture flexible high frequency diodes by only using lamination at room temperature, without the requirement of any printing equipment, is demonstrated. To the best of our knowledge, this is the first time cellulose is used to produce a free-standing composite semiconducting film which can be utilized in the fabrication of electronic devices operating in the GHz range. Thanks to the self-adhesive properties of the NFC:Si-μP film the diode stack can be delaminated from the Al electrode and subsequently transferred to yet another substrate, hence, reconfigurable GHz-operating diode circuits are enabled.

## Results

### Properties of the NFC:Si-μP film

**Figure 1:**
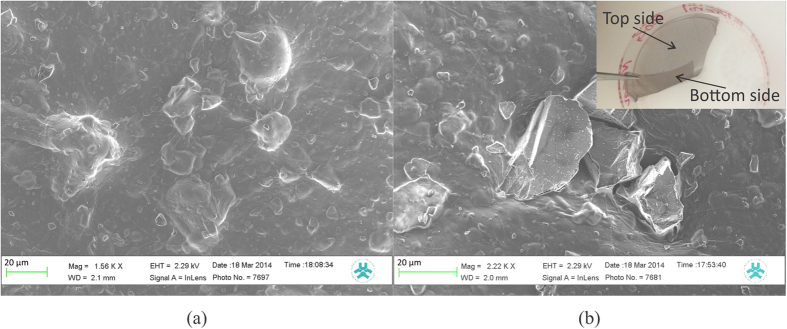
The inset in the upper right corner shows a self-supporting NFC:Si film that is being removed from the Petri dish, while (**a**,**b**) are the SEM images from the top and the bottom surfaces of the film, respectively.

The NFC:Si-μP films are entirely self-supporting and semitransparent, and can easily be peeled off from the petri dish by using ordinary tweezers, as demonstrated in the inset of Fig. 1. The film is slightly sticky and exhibits self-adhesive surface properties due to the presence of glycerol. The bottom surface, i.e. the side that is in contact with the Petri dish surface during drying, is relatively more adhesive than the top surface. This probably indicates that there is a slightly higher glycerol content at the bottom section of the film as compared to the top volume. SEM was used to study the morphology and microstructure of both the top (Fig. 1a) and the bottom (Fig. 1b) surfaces of the NFC:Si film. SEM images show that although the sieve used to filter the Si particles is 100 μm, the crossing line lengths of the largest particles are shorter than 50 μm.

The size of the particles and the proportions of NFC, Si-μPs and glycerol in the films are chosen so that the larger micro-particles extend from the top to the bottom surfaces of the film. This gives that only one layer of large-sized Si-μPs provide the electrical current pathway of the diode. This in turn limits the number of interface layers that charges need to pass. The NFC matrix has a thickness of 10–15 μm according to the optical profilometer measurements. This thickness makes it possible for the film to be self-supporting and also robust enough to be handled in the following process steps using peeling, transfer and lamination. The SEM images show that all Si-μPs are covered with NFC along the top surface. This NFC top-layer is probably very thin for the larger Si-μPs that are extending outside the NFC film matrix; while at the bottom surface many of the particles are not covered by NFC. The SEM images taken from the top surface of the films before and after they are pressed using the calender machine suggest that the mechanical pressure during calendering might cause the thin NFC layer to rupture, and thereby ensuring good electrical contact between the Si-μPs and the electrodes.

### Device structure and mechanism

**Figure 2:**
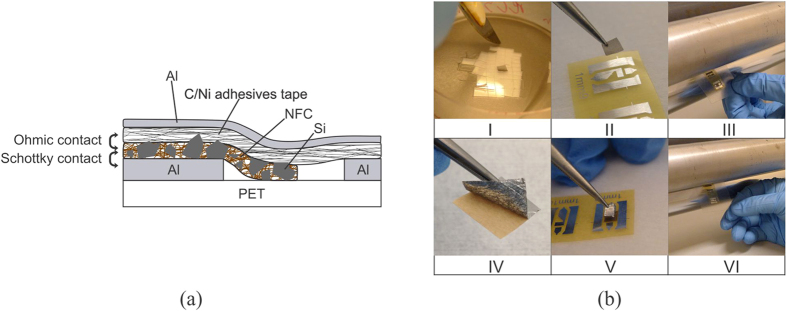
(**a**) The structure of the final device. The scale is schematic and does not correlate to the real dimensions. (**b**) The fabrication process of the diode: Peeling off the Si film (I); Attaching it to the substrate (II); Calendering (III); Peeling off the Ni/C double side adhesive tape (IV); Attaching the Ni/C tape to the Si film (V); Calendering once more (VI).

A schematic cross-section view of the resulting NFC:Si-μP diode structure, together with the manufacturing process is given in Fig. 2. The Al electrode that is in direct contact with the NFC:Si-μP film is the anode and the Ni/C tape is the cathode in the diode structure. The Si-μPs embedded in the NFC film form Schottky contacts to the Al bottom layer (see Supplementary information section 1)^18^. The nature of the contact between a NFC:Si-μP film and the Ni/C tape electrode is ohmic. This was confirmed by I-V measurements performed on a symmetric structure of the film sandwiched between two layers of Ni/C tape (see Supplementary information section 1). Ni has been used to create ohmic contacts to Si in the past^30^, however to the best of our knowledge, this is the first time a double adhesive conductive Ni plated carbon fiber tape is used as the ohmic contact for flexible GHz diodes. One of the prime advantages of using this kind of contact electrodes over the common conductive inks, such as carbon paste, is that the conductive tape cannot penetrate into, or even through, the semiconducting layer to cause short circuits down to the bottom contact. Another function of the Ni/C tape is that it spans over the junction area and attaches to the PET substrate, i.e. it ensures a mechanically stable diode that is kept in place. The use of an extra Al foil on top of the Ni/C tape enhances the lateral conductivity of the top contact, which bridges between the top side of the semiconducting film and the second electrode on the substrate.

### Diode performance and modeling

**Figure 3:**
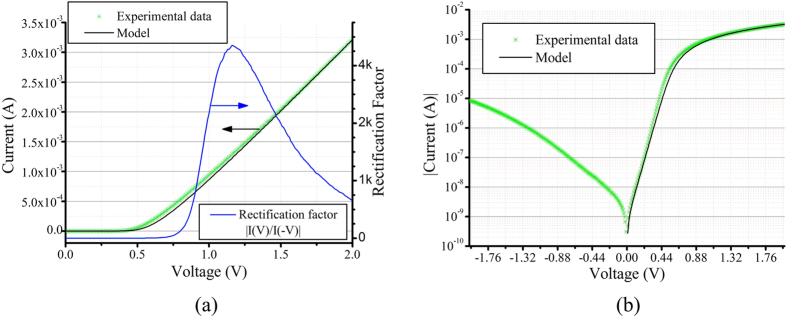
(**a**) The rectification ratio and the I-V plots of the diode (green) and the model (black) in linear scale and (**b**) logarithmic-linear plots of the I-V curve of the diode (green) and the model (black).

The I-V curve of the NFC:Si-μP diode is shown in Fig. 3. In all the measurements and modeling the current level is the preferred description of the charge transport through the device over the current density. This is because the semiconducting layer of the diode is heterogeneous and consists of multiple particles, which together contribute in parallel to the resulting measured current. In addition, each particle contributes differently to the electrical conduction depending on its shape, orientation, size, etc.; therefore, the current density would not be an accurate term to use for this device structure. In all the following results the overlapping surface area between the two electrodes sandwiching the NFC:Si-μP film is 2 mm^2^.

The device has a sharp turn-on voltage at around 0.5 V, and a rectification ratio up to 4 × 10^3^ is achieved below 2 V bias. This indicates that a good Schottky is established between the Al bottom electrode and the Si particles. At higher voltages the leakage current increases, resulting in lower rectification factor. The relatively high leakage current is probably due to the Schottky barrier lowering and defect levels on the surface of the particles^31^. However, the diode performance is still sufficient in many of the low-power (mW range) energy harvesting applications. Within a batch of six samples, two have a current level on the order of 10^−5^ A, two have about 10^−3^ A and two have 10^−2^ A of forward current at 2 V. The samples with the 10^−5^ A level are discarded due to their low current. All of the six samples in the batch have rectification factor of up to 100–1000 below 2 V bias. The current-voltage relationship of a Schottky diode can be expressed as^32^:


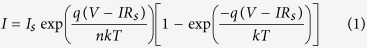


where *R*_*s*_ is the series resistance, *n* is the ideality factor of the diode and *q, k* and *T* are the elementary charge, the Boltzmann constant and the absolute temperature, respectively. *I*_*s*_ is the reverse current that is dependent on the contact barrier height (*φ*_*b*_), the contact surface area (A), the Richardson constant of the semiconductor (A*) and the temperature and can be expressed as^33^:


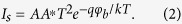


The ideality factor, which equals 1 for an ideal diode, is an indication of the deviation of the diode behavior from the thermionic emission mechanism in an ideal Schottky contact^32^. The y-axis intercept of the linear part of the logarithmic-linear plot of the I-V curve gives the reverse current and the ideality factor can be calculated using the slope of the same curve. The reverse current and the ideality factor calculated using this method are 6.91 × 10^−10^ A and 1.8, respectively. By using the method suggested by Cheung *et al*.^34^,^35^, the series resistance and the ideality factor can be obtained from a plot of *dV/d*(*ln*(*I*)) vs. *I* (see Supplementary information Fig. 2). Using Cheung’s method, the series resistance is calculated to be 428 Ω, and the value obtained for the ideality factor is 1.62, which is very close to the value extracted from the logarithmic-linear plot of the I-V curve. As illustrated in Fig. 3, the model including these parameters (the solid line) shows a good fit to the experimental data (green stars). In order to estimate the barrier height the I-V curve measurement is repeated in a temperature range between −5 °C and 50 °C. A barrier height of 0.35 eV is then extracted from the slope of the Richardson plot (see Supplementary information).

Furthermore, the possibility of reconfiguring the sticker label diode was successfully tested. It was possible to lift off the upper part of the diode consisting of the NFC:Si-μP film laminated with the Ni/C conductive tape. This part of the diode stack was then transferred to another substrate, and after an additional calendering process step a new diode with similar I-V performance, as compared to the initial diode, was obtained.

**Figure 4:**
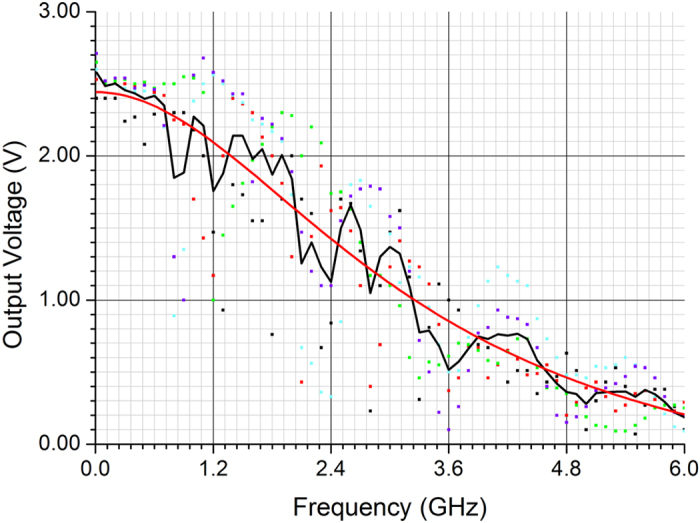
The frequency response of a diode device. The input power is 77 mW and the measurement load is a 1 MΩ resistance in parallel with a 13 pF capacitance.

Since energy harvesting and AC to DC conversion are among the main applications that are considered for this type of flexible diode, it is important to characterize the range of operating frequencies of the diode. This is verified by the frequency response of the diode, which is characterized by applying a single harmonic signal, and measuring the output DC voltage while sweeping the input frequency. At high frequencies the input signal at each node in the measurement circuit (see Supplementary information Fig. 4) can be reflected back and forth if the input and output impedance are not matched. This results in an increase or a decrease of the output signal depending on the phase difference between the reflected and the incoming wave. The phase difference between the two signals depends on different parameters such as input frequency, load, cable lengths etc. This effect appears as fluctuations in the output DC level as the frequency is varied, and consequently makes it difficult to estimate the cut-off frequency. To resolve this problem the frequency response is characterized several times while applying small changes in the measurement setup such as using cables with different lengths, and by connecting RC loads and a 1 dB attenuator to the nodes to mitigate the reflected signal. These small changes in the setup cause the DC level to have less fluctuation in some frequency ranges, though it does not resolve the problem of fluctuations across the entire bandwidth. An averaging approach is used to estimate the cut-off frequency of the device, which is defined as the frequency where the output power drops to half (or equivalently the voltage drops to 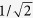) of the corresponding value at the lowest frequency (10 MHz in our measurement). The frequency response of a representative diode, illustrated in Fig. 4, shows a cut-off frequency of 1.8 GHz even though the diode still produces a DC output voltage greater than 1 V at 3 GHz. The applied input power is 18.9 dBm (77 mW, after subtracting the damping effect from the attenuator), and this is a power that can be supplied for example by a mobile phone that is held in close proximity to the diode while making a call within the GSM band^36^. Among a batch of six devices, two of them have a cut-off frequency above 1 GHz. However, all of the devices have at least a cut-off frequency of 100 MHz.

Although overlapping surface area of the two electrodes sandwiching the NFC:Si-μP film is 2 mm^2^, the surface area of the Schottky contact is much smaller since only a fraction of the surface area of each particle is in contact with the Al substrate. According to work by Champlin *et al*.^37^, Schottky diodes with a contact diameter of 20 μm can reach a cut-off frequency of 10 GHz; and even with a contact diameter of 100 μm it is still possible to achieve a cut-off frequency of 1 GHz.

The output power for a resistive load is measured at two fixed frequencies, 0.9 GHz and 1.8 GHz, which correspond to the two GSM frequencies, by using the same set-up and input power as in the frequency response measurement. A variable load resistor is connected to the circuit and the output power is measured while the load resistance is varied between 50 Ω to 1 MΩ at a constant input power of 77 mW. As the load resistance is increased, the voltage drop across the load is also increased while the current simultaneously drops. The power, which is the product of current and voltage, is low as long as the resistance is very low (i.e., close to short circuit), since the voltage drop is small. Adjusting the load resistance to a very high value (i.e., close to open circuit) also results in a low output power since the current is negligible. On the contrary, the output power has a peak when the resistance of the load is equal to the real part of the output impedance, which for this diode appears to be 800 Ω and 1400 Ω at the input frequencies of 0.9 MHz and 1.8 MHz, respectively (Supplementary information Fig. 5). The maximum output power available with the mentioned input is around 1.26 mW at 0.9 GHz and 0.25 mW at 1.8 GHz. Although these values are relatively low as compared to commercial diodes^2^, they are sufficient for powering low input power devices such as printed organic electrochromic displays and sensors^18^. It should be noted that the maximum output power is reached only when the impedance of the load matches the output impedance of the entire measurement circuit, including the diode, which is not purely resistive^38^.

## Discussion

To summarize, NFC has successfully been used as a matrix and binder material to manufacture self-adhesive and self-supporting Si-μP composite films. By using Si particles of up to 50 μm in size, it is possible to obtain mechanically stable NFC:Si-μP films with a base thickness of 10–15 μm, from which large Si-μPs are extending outside the NFC binder layer.

A diode structure was created by incorporating the NFC:Si-μP film between Al/PET and a conductive tape by using a simple “peel and stick” assembly method. Schottky junctions are formed between the Si particles and the Al bottom electrode, while the ohmic contact is ensured by a double adhesive conductive tape based on Ni plated carbon fibers.

The entire process of fabricating both the semiconducting Si-μP composite film and the diode is performed at room temperature. The only machine used in this process is a calender machine that presses and laminates the different layers/films together.

To our knowledge, for the first time a very simple lamination based method is used to produce a free-standing semiconducting composite film that can be implemented as the semiconductive layer in flexible semiconducting devices with GHz operation frequency. The slight stickiness of the NFC:Si-μP film (due to the presence of glycerol in the film) ensures good adhesion upon lamination. Furthermore, the use of the Ni/C conductive tape eliminates the need for a printing process and basically excludes the risk for the top and bottom contacts to short-circuit the semiconducting layer. Efforts were not made in this paper to optimize sample-to-sample reproducibility. We expect, however, that the most significant source of sample variation is the Si particle size distribution, and an improved sorting procedure would lead to much smaller variation.

The method presented here opens up the possibility to manufacture a diode on a smart label by a simple method, and the diode can for example be combined with other “sticker-label” electrochemical components^29^ to create wirelessly powered smart labels containing electrochromic displays, logic circuits based on electrochemical transistors and also sensors. It can be envisioned that such easy-to-assemble and self-adhesive “sticker-label” electronic devices could facilitate a future functional *e-*label technology that can be connected to existing IoT circuit platforms and networks, enabling ubiquitous data collection, swift information exchange, and distributed diagnostics.

## Methods

### Material preparation

For preparation of Si-μPs, a single crystal Si wafer with a resistivity of 0.01–0.02 Ω·cm and doped with Sb is used. First, the wafer is crushed and then milled for 2 hours in a Retsch PM100 ball milling machine. The obtained particles are further fractioned using a Retsch sieve machine with a 100 μm stainless steel sieve, where passed μPs are collected and used for the diode manufacturing process.

An aqueous dispersion of anionic NFC gel was prepared by high-pressure homogenization of carboxylethylated cellulose fibers and consecutively by an ultrasonication centrifugation process at Innventia AB^27^.

### Preparation of the film

The fractioned Si powder is then mixed with 0.5 wt% NFC water suspension and glycerol; the latter ingredient is included to enhance the surface adhesion of the dried film. The mixture is then further diluted with water by a factor of two and thoroughly mixed with a shear mixer for 3 minutes, and then further mixed with an ultrasonic gun (Sonopuls 2200) by applying 1 s pulses, at a 10% duty cycle, with 20 W power for 30–60 s. 5 g of the final mixture is cast into a petri dish with a diameter of 50 mm and is then left to dry in ambient environment. The resulting dried film contains 12.5 wt% Si-μPs, 51.5 wt% glycerol and 36 wt% NFC. The resulting films can easily be peeled off from the bottom of the Petri dishes. The surfaces of the two sides of the NFC:Si film were characterized by optical profilometer and scanning electron microscope (SEM).

### Device fabrication

The diode structure is simply fabricated by laminating and pressing the different layers of materials together. The only equipment used is a calender machine working at room temperature. A flexible substrate composed of 9 μm Al foil laminated onto a 36 μm PET foil was used. The Al substrate is patterned by a photolithography process, where the pattern was defined and etched by photoresist and buffered phosphoric acid, respectively. The Al substrate can also be patterned via dry-phase milling to skip the photolithography steps, however this method has not been used for the for the UHF devices reported here^39^. A piece of the NFC:Si-μPs composite film is then cut and pasted onto the 1 mm wide Al strip on the pre-patterned Al substrate. The structure is then pressed using a calender machine, with a pressure of 3 bar, which ensures good contact between the Al strip and the NFC:Si-μP film. A double adhesive conducting tape consisting of Ni plated carbon fibers (3 M 9713) (Ni/C tape hereafter), supported with a layer of Al foil on top, is then applied on top of the NFC:Si-μPs layer. The entire PET/Al/NFC:Si-μP/Ni/C stack structure is pressed once more using the calender machine, again with a pressure of 3 bar. The overlapping surface area of the Si film and the top electrode is 2 mm^2^.

### Device characterization

DC characteristics of the diodes were measured by a Keithley 4200-SCS Semiconductor Characterization System.

The high frequency performance of the diodes is characterized by applying a single harmonic signal, and the output DC voltage is then measured within a span of frequencies ranging from 10 MHz to 6 GHz using the set-up illustrated in Supplementary information Fig. 4 18. An Agilent RF frequency signal generator 8665B is connected to the sample via a 50 Ω cable and a microwave Air Coplanar Probe (ACP) from Cascade Microtech (custom made with 1250 μm pitch between ground and signal probe tips), and the output signal of the sample is also probed with an ACP and connected with a 50 Ω cable to an oscilloscope (Tektronix TDS3034) with the input impedance of 1 MΩ resistance in parallel with a 13 pF capacitance. The sample was connected to an input power of 19.9 dBm via a 1 dB attenuator in order to reduce signal reflections. To measure the power harvesting capability of the diode, a set-up similar to the high frequency measurement is used, but a resistive load is connected in series to the diode instead of the oscilloscope. The voltage drop across the resistive load is measured at input frequencies of 0.9 and 1.8 GHz while the load is varied between 50 Ω and 1 MΩ.

## Additional Information

**How to cite this article**: Sani, N. *et al*. Flexible Lamination-Fabricated Ultra-High Frequency Diodes Based on Self-Supporting Semiconducting Composite Film of Silicon Micro-Particles and Nano-Fibrillated Cellulose. *Sci. Rep.* **6**, 28921; doi: 10.1038/srep28921 (2016).

## Supplementary Material

Supplementary Information
