# Supplementary material for: Flexible Lamination-Fabricated Ultra-High Frequency Diodes Based on Self-Supporting Semiconducting Composite Film of Silicon Micro-Particles and Nano-Fibrillated Cellulose
Source: Sci Rep. 2016 Jun 30;6:28921. doi: 10.1038/srep28921 (PMC4928109; doi:10.1038/srep28921)
Supplement: Supplementary Information [file srep28921-s1.pdf]

Flexible Lamination-Fabricated Ultra-High Frequency Diodes Based on Self-Supporting Semiconducting Composite Film of Silicon Micro-Particles and Nano-Fibrillated Cellulose

Negar Sani[1], Xin Wang[2*], Hjalmar Granberg[3], Peter Andersson Ersman[2], Xavier Crispin[1], Peter Dyreklev[2], Isak Engquist[1*], Göran Gustafsson[2] and Magnus Berggren[1]

[1]Department of Science and Technology, Linköping University, SE-601 74 Norrköping, Sweden

[2]Printed Electronics, Acreo Swedish ICT AB, Box 787, SE-601 17 Norrköping, Sweden

[3]INNVENTIA AB, Box 5604, SE-114 86 Stockholm, Sweden

Supplementary Information

1. Identifying the rectifying and ohmic contacts

In order to identify the type of contact each electrode makes with the NFC:Si film, two structures are examined. The first structure consists of the NFC:Si film with C/Ni tape (conductive adhesive tape consisting of Ni coated carbon fibers) as the top and the bottom contacts and the second structure consists of the NFC:Si film with Al as the top and the bottom contacts. The I-V curves of the two structures are illustrated in Supplementary Figure 1. The linearity of the I-V curve in Supplementary Figure 1(a) indicates that the contact between the Si in the NFC:Si film and the C/Ni tape has a low, or no, energy barrier for the charge carriers. On the other hand, the low current amplitude of the I-V curve in Supplementary Figure 1(b) shows that there is a high energy barrier for the charge carriers for both polarities of the applied voltage. Therefore, the contact between the Si and the C/Ni tape is ohmic, while the rectifying Schottky contact is provided by the energy barrier between the Al and Si.

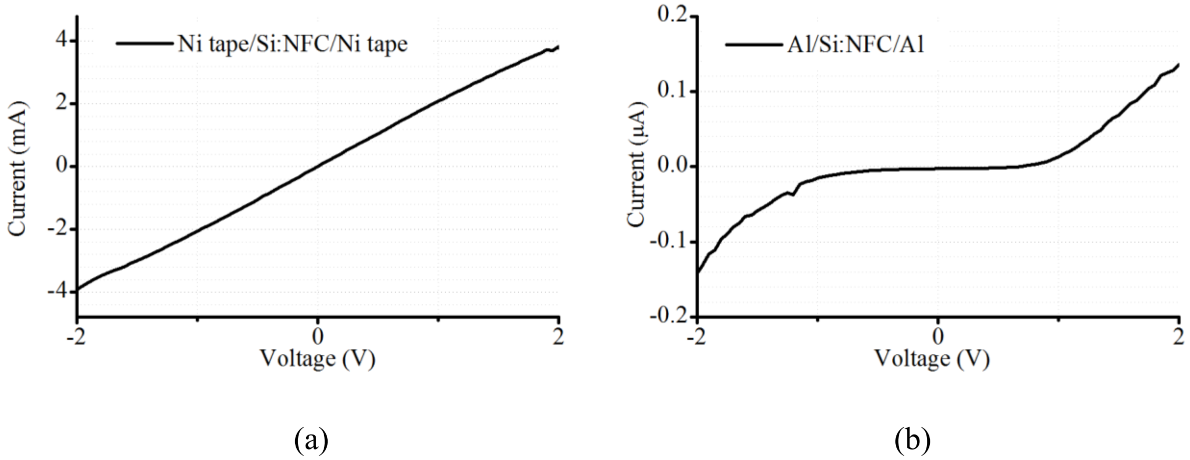

Supplementary Figure 1. (a) The I-V curve of the NFC:Si film with C/Ni tape as the top and the bottom contacts. (b) The I-V curve of the NFC:Si film with Al as the top and the bottom contacts.

2. Modeling

The series resistance and the ideality factor can be obtained from a plot of *dV/d(ln(I))* vs. *I* according to a method suggested by Cheung et al. [1,2]:

$$\frac{dV}{d(\ln I)} = n\frac{kT}{q} + IR_s \quad (1)$$

A plot of *dV/d(ln(I))* vs. *I*, illustrated in Supplementary Figure 2, has a slope of R_s and a y-axis intercept of nkT/q. The values calculated for R_s and n using this method are 428 Ω and 1.62 respectively.

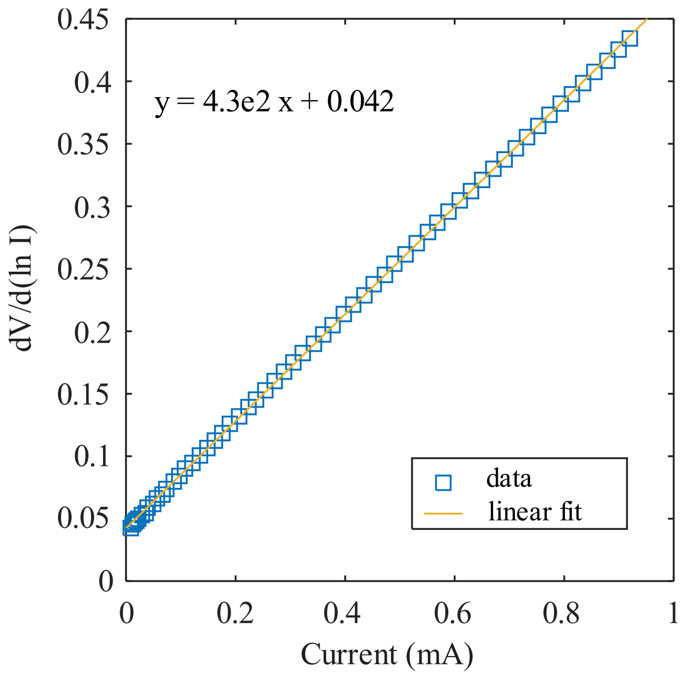

Supplementary Figure 2. The plot of *dV/d(ln(I))* vs. *I*.

One of the common methods to estimate the contact barrier height is to use the semilogarithmic plot of I/T^2 vs. $1/T$ at a certain voltage bias. For V ≫ kT/q, the Schottky temperature-current relation can be written as [3]:

$$\ln(I/T^2) = \ln AA^* - q(\varphi_b - V/n)/kT \quad (2)$$

The plot of I/T^2 vs. $1/T$, which is frequently referred to as the Richardson plot, has a slope of $q(\varphi_b - V/n)/kT$. The Richardson plot of the diode for a temperature range between -5° C and 50° C, at V=0.2 V, is illustrated in Supplementary Figure 3. The slope of the line is 1.2, which gives a barrier height of 0.35 eV.

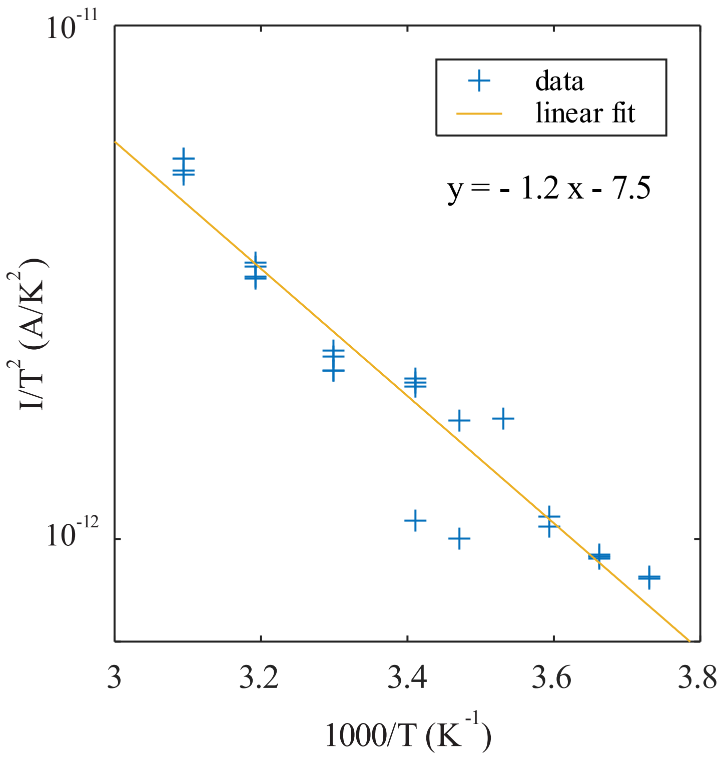

Supplementary Figure 3. The Richardson plot of the diode.

3. The measurement setup for frequency response measurements

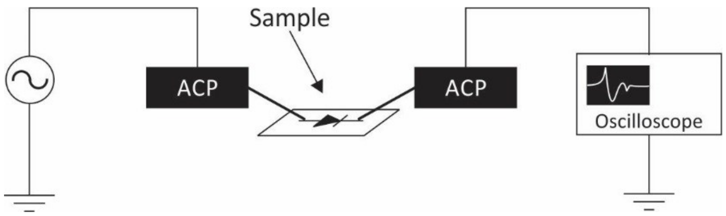

Supplementary Figure 4. The measurement setup for frequency response measurements.

4. The output power of the diode

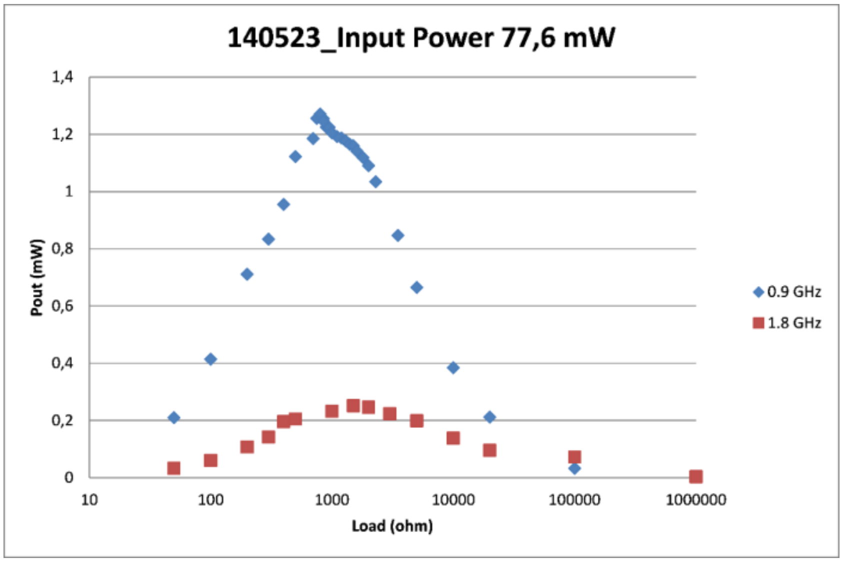

Supplementary Figure 5. The output power as a function of resistive load, where the input frequencies were 0.9 GHz (blue) and 1.8 GHz (red)

References

1 Ayyildiz, E. *et al.* Effect of series resistance on the forward current-voltage characteristics of Schottky diodes in the presence of interfacial layer. *Solid-State Electronics* **39**, 83-87 (1996).

2 Cheung, S. K. & Cheung, N. W. Extraction of Schottky diode parameters from forward current-voltage characteristics. *Applied Physics Letters* **49**, 85-87 (1986).

3 Schroder, D. K. *Semiconductor Material and Device Characterization*. (Wiley, 2006).
